# Pivotal avenue for hybrid electron transport layer-based perovskite solar cells with improved efficiency

**DOI:** 10.1038/s41598-023-33419-1

**Published:** 2023-11-09

**Authors:** Poonam Subudhi, Deepak Punetha

**Affiliations:** 1 School of Advanced Sciences, Vellore Institute of Technology (VIT) University, Chennai, Tamilnadu 600127 India; 2 School of Electronics Engineering, Vellore Institute of Technology (VIT) University, Chennai, Tamilnadu 600127 India; 3 Department of Electronics & Communication Engineering, Motilal Nehru National Institute of Technology (MNNIT), Allahabad, Prayagraj, Uttar Pradesh 211004 India

**Keywords:** Energy science and technology, Engineering, Materials science, Nanoscience and technology

## Abstract

This study conducted a simulative analysis of different hybrid perovskite solar cells with various hybrid electron transport layers (ETL) and hole transport layers (HTL). The electron transport layer boosts durability, lowers production costs, increases stability, improves light absorption, and increases efficiency. Hybrid ETLs are taken into consideration to improve the device's performance. The selected hybrid ETLs (PCBM–SnS_2_, TiO_2_–SnO_2_, and PCBM–PCPB) were modeled with four hybrid perovskite absorbers (CsPbI_3_, FAPbI_3_, MAPbI_3,_ and FAMAPbI_3_) and five HTLs (PEDOT: PSS, CuI, Spiro-OMeTAD, CBTS, and NiO). Three sets of solar cells are found to be the most effective configurations after investigating over sixty different combinations of perovskite solar cell architectures. The structures show CBTS as the efficient HTL for FAMAPbI_3_ with all three hybrid ETLs. Besides, a holistic analysis of the effect of several factors such as the defect density and thickness of the absorber layer, temperature, parasitic resistances, capacitance, Mott–Schottky, impedance, conduction band offset, and current density–voltage and quantum efficiency characteristics is performed. The results show a maximum power conversion efficiency of 25.57%, 26.35%, and 23.36% with PCBM–SnS_2_, TiO_2_–SnO_2_, and PCBM–PCPB respectively. Among the studied hybrid ETLs, perovskite solar cell associated with TiO_2_–SnO_2_ has depicted a superior performance (Voc = 1.12 V, Jsc = 26.88 mA/cm^2^, FF = 87.27%). The efficiency of the perovskite solar cell using this study has been drastically enhanced compared to the previous experimental report. The proposed strategy provides a new avenue for attaining clean energy and allows researchers to pave the way for further design optimization to obtain high-performance solar cell devices.

## Introduction

**Figure 1:**
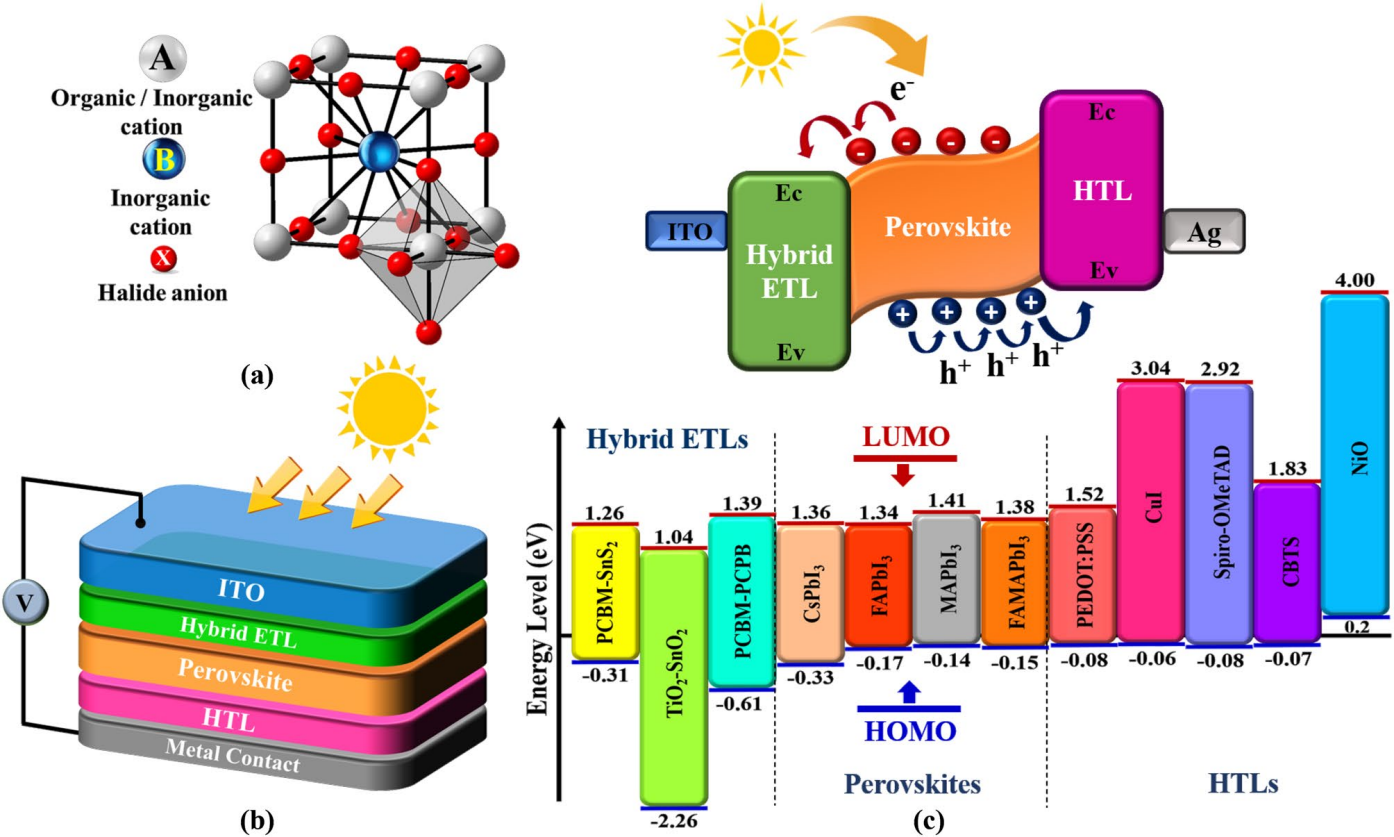
(**a**) A 3D cubic crystal structure of an ideal perovskite with ABX_3_ general formula, (**b**) schematic of the inverted perovskite solar cell device: ITO/Hybrid ETL/Perovskite/HTL/Metal contact and (**c**) schematic representation of the transformation of the charge carriers with simplified flat band energy level diagram of PSC modelled and investigated in this work.

The need for environmentally friendly and renewable energy sources to meet the rising demand for energy from the expanding population and industry is one of the main issues facing our society today. A widely anticipated technology is solar cell technology, which is anticipated to be effective in generating clean energy at a low cost and with minimal pollution^1,2^. Solar cells use a technique known as photovoltaics to convert sunlight into electrical power. As a result, they are considered a clean and renewable energy source because they do not emit any pollutants or greenhouse gases when in use^3^. Perovskite solar cells now come in a variety of forms, making them a desirable choice for meeting energy needs. A potential new technology in the emerging area of photovoltaics is perovskite solar cells. Due to its potential for high power conversion efficiency and affordable production, it has recently attracted a lot of interest^4^. Perovskite materials are adaptive and flexible for solar cell systems due to their distinctive crystal structure, which combines organic and inorganic elements^5^. Perovskite solar cells also have the advantages of being lightweight, flexible, having a direct band gap energy, better spectrum responsiveness, and ease of fabrication at room temperature. The typical chemical formula for perovskite is ABX_3_, where A is an enormous organic/inorganic cation that is surrounded by smaller anions X (halogen ions: Br, I, F, and Cl), which are grouped in a cubic configuration, with a smaller B inorganic cation in the middle, as illustrated in Fig. 1a. Perovskites are divided into two groups based on their elemental composition: organic–inorganic hybrid perovskites (OIHP) and metal halide perovskites (all-inorganic perovskite)^6,7^. PbBiX_3_, CsPbX_3_, and other inorganic halide perovskite-based solar cells all have excellent thermal stability, good chemical stability, and high transparency, making them desirable materials for usage in challenging situations, long-term applications, and optical devices^8^. However, organic–inorganic hybrid perovskites, such as MAPbX_3_, FABiX_3_, and EASnX_3_, tend to be more adaptable and can be processed at a lower cost, making them a more widely used and accessible material. OIHPs are a potential material for application in photovoltaics because of their additional advantages, which include a configurable bandgap, long carrier diffusion length, velocity, weak binding energy, big Bohr radius, high dielectric constant, and better optoelectronic properties^9^. Perovskite materials rose to the top of the list of options for making inexpensive, highly effective solar cells as a result of all these benefits.

To attain optimal efficiency, perovskite solar cells typically have an absorber layer (made of perovskite material) sandwiched between an electron transport layer (ETL) and a hole transport layer (HTL). ETL and HTL are n- and p-type materials, respectively. When the absorber is exposed to light, the HTL enhances the hole extraction while the ETL extracts the photoelectrons produced by the absorber material^10^. The structure of PSCs is categorized as p-i-n when the p-type carrier is positioned on the substrate and n-i-p when the n-type is positioned, depending on the type of charge-transporting layers on the substrate^11,12^. The nature and types of these layers substantially impact the stability and functionality of the device. The n-i-p type is the more effective of the two structures. When selecting an ETL for perovskite solar cells, factors including cost, conduction band offset between the absorber and ETL, and compatibility with other layers' high electron mobility are taken into account. Due to their superior electrical characteristics and strong chemical stability, colloidal ETLs, including TiO_2_, PCBM, and SnO_2_ were often employed by researchers. To further improve PSC performance, a hybrid ETL is formed, adding qualities like high electron mobility, outstanding thermal stability, and good interface quality, which leads to the long-term stability of the concerned device^13–16^. Considerations are made for an HTL's valence band offset and hole mobility between the absorber layer and HTL. Perovskite solar cells frequently employ HTLs such as PEDOT: PSS, CBTS, and Spiro-OMeTAD because of their superior processability and tunability. NiO, CuO, CuI, and CuO_2_ are just a few of the inorganic metal compounds that are used as the HTL in PSCs^17^. Simulation studies and computer modelling are essential for identifying suitable materials for the various layers of perovskite solar cells since it is expensive and time-consuming to fabricate the different levels of a multilayered perovskite solar cell through experimentation. The different device parameters are analyzed using the solar cell simulator to do this.

In the current work, a simulation comparison analysis was conducted for four lead-based absorbing materials used in solar cells: CsPbI_3_, FAPbI_3_, MAPbI_3_, and FAMAPbI_3_. In addition, the hybrid electron transporting layer was composed of PCBM–SnS_2_, TiO_2_–SnO_2_, and PCBM–PCPB, while the hole transfer layer was composed of PEDOT:PSS, CuI, Spiro-OMeTAD, CBTS, and NiO. To maximize the effectiveness of the hybrid ETL-based lead perovskite solar cell, the major goal of the current study is to analyze the impact of various variables on the PSC and, as a result, optimize the solar cell parameters. The effect has been studied in terms of absorber layer thickness, defect density, device temperature, and resistances, including sheet and shunt resistance. The hybrid ETLs have enhanced the performance of the perovskite active material FAMAPbI_3_ using CBTS as HTL. The conduction band offset, capacitance, generation/recombination rate, impedance (recombination resistance), J–V Characteristics, and quantum efficiency of these optimized device architectures are also computed.

## Methodology and device parameters

### A Numerical simulation

Improvements in materials, construction, contact systems, and characterizations have led to an increase in solar cell efficiency. The efficiency of solar cells may also be optimized by modeling and analysis. The foundations of solar cells are simpler to understand when they are modeled numerically, and the main variables affecting their performance are revealed. The calculations were carried out using SCAPS-1D software (Solar Cell Capacitance Simulator One Dimension). In addition to computing several profiles, such as grading, generation, recombination, carrier transport method, and device architectural faults, this simulation tool also analyses the physics of the model^18^. It has previously been used to simulate several types of perovskite solar cells. The simulation approach of this tool is based on the solution of one-dimensional equations governing semiconductor materials. The equations are essentially linked continuous differential equations with electron and hole derivatives from Poisson equations^19^. The following is the Poisson equation that describes the connection between the space charge density and the electric field of a p–n junction^5^:

1 $$\frac{dE}{dx}= \frac{d}{dx}\left(-\varepsilon (x)\frac{d\psi }{dx}\right)=q\left[p\left(x\right)-n\left(x\right)+{N}_{D}^{+}\left(x\right)- {N}_{A}^{-}\left(x\right)+ {\rho }_{t}\left(x\right)- {n}_{t}(x)\right]$$

where *E* is the electric field, *ε* is the electrostatic potential, n is the total electron density, *ψ* is the dielectric constant (permittivity), *q* is the electron charge, *p* is the total hole density, ${N}_{A}^{-}$ is the ionized acceptor-like doping concentration, ${N}_{D}^{+}$ is the ionized donor-like doping concentration, ${\rho }_{t}$ is the trapped hole concentration, ${n}_{t}$ is the trapped electron concentration, and x is the one-dimensional position coordinate.

The following are the continuity equations for electrons (Eq. 2) and holes (Eq. 3):

2 $$\frac{\partial {j}_{n}}{\partial x}=q \left({R}_{n}-G+ \frac{\partial n}{\partial t}\right)$$

3 $$\frac{\partial {j}_{p}}{\partial x}=q \left({R}_{p}-G+ \frac{\partial p}{\partial t}\right)$$

Here, ${R}_{n}$ and ${R}_{p}$ are the net recombination rates for electron and hole per unit volume, respectively, ${j}_{n}$ and ${j}_{p}$ are the electron and hole current densities, and *G* is the generation rate per unit volume. This simulation tool solves the above equations under steady-state conditions.

### Device architecture and simulated parameters

Using the solar cell simulator software, we modelled the perovskite solar cells' electrical and physical characteristics for this research. In the simulation, the device structure is an inverted planar (n-i-p) type made up of back contact, ITO, hybrid electron transport layer (Hybrid-ETL), perovskite layer, and hole transport layer (HTL). Figure 1b depicts the architecture of the device. The absorber layer was composed of CsPbI_3_, FAPbI_3_, MAPbI_3_, and FAMAPbI_3_, and the HTL over the back contact was composed of PEDOT:PSS, CuI, Spiro-OMeTAD, CBTS, and NiO, and the hybrid ETL was composed of PCBM–SnS_2_, TiO_2_–SnO_2_, and PCBM–PCPB. These ETL and HTL materials are chosen due to their suitable band edges that match the conduction band minimum and valence band maximum of absorber materials to obtain a high-efficiency solar cell. The schematic diagram of bandgap alignment for different ETL and HTL materials with respect to absorber layers is shown in Fig. 1c. The highest occupied molecular orbital (HOMO) levels for all the HTL materials are well aligned with the valence band level of absorber materials, and it has higher values to ensure the process of photogenerated hole extraction. Whereas the lowest unoccupied molecular orbital (LUMO) level of ETL is well aligned with the conduction band of the absorber materials to accept electrons ^20^. Using these materials, the simulation is executed using an incident power of 1000 W/m^2^ and the AM1.5 Global spectrum. The remaining simulation variables are left at their normal settings, and the operating temperature is set at 300 K. The parameters found in the literature are utilized for the simulations of the designed structure. Tables 1 and 2 provide the key parameters for the hole transporting and perovskite absorber layers, respectively. The majority of the parameters for hybrid ETLs are expected to have the same properties as those that were gleaned from the literature on experimental work. Table 3 displays the optimized variables, and Table 4 provides the parameters for interface layers. A more realistic perovskite solar cell is simulated by including a hybrid ETL/Perovskite interface layer and Perovskite/HTL interface layer.

**Table 1:** The set parameters for different lead-based hybrid perovskites^21–26^.

Perovskites	CsPbI_3_	FAPbI_3_	MAPbI_3_	FAMAPbI_3_
Thickness (nm)	400	350	900	550
Band gap, *E*_*g*_ (eV)	1.694	1.51	1.55	1.53
Electron affinity, χ (eV)	3.95	4	3.9	4
Dielectric permittivity (relative), ε_r_	6	6.6	30	9
CB effective density of states, *N*_*C*_ (1/cm^3^)	1.1 × 10^20^	1.2 × 10^19^	2 × 10^18^	1 × 10^19^
VB effective density of states, *N*_*V*_ (1/cm^3^)	8 × 10^19^	2.9 × 10^18^	2 × 10^19^	5 × 10^18^
Electron mobility, ${\mu }_{n}$ (cm^2^/ V-s)	25	2.7	10	5
Hole mobility, ${\mu }_{e}$ (cm^2^/ V-s)	25	1.8	10	3
Shallow uniform acceptor density, *N*_*A*_ (1/cm^3^)	–	1.3 × 10^16^	1 × 10^17^	2 × 10^16^
Shallow uniform donor density, *N*_*D*_ (1/cm^3^)	1 × 10^15^	1.3 × 10^16^	–	2 × 10^16^
Total defect density, *N*_*t*_ (1/cm^3^)	1 × 10^14^	1 × 10^14^	1 × 10^14^	1 × 10^13^
Thermal velocity of electrons and holes (cm/s)	1 × 10^7^	1 × 10^7^	1 × 10^7^	1 × 10^7^
Capture cross section of electrons and holes (cm^2^)	2 × 10^–14^	2 × 10^–14^	2 × 10^–14^	2 × 10^–14^
Absorption coefficient, α (cm^−1^)	1 × 10^5^	1 × 10^5^	1 × 10^5^	1 × 10^5^

**Table 2:** Parameters for various hole transporting layers^7,12,27,28^.

HTL	PEDOT:PSS	CuI	Spiro-OMeTAD	CBTS	NiO
Thickness (nm)	50	100	200	100	40
Band gap, *E*_*g*_ (eV)	1.6	3.1	3	1.9	3.8
Electron affinity, χ (eV)	3.4	2.1	2.2	3.6	1.46
Dielectric permittivity (relative), ε_r_	3	6.5	3	5.4	10.7
CB effective density of states, *N*_*C*_ (1/cm^3^)	2.2 × 10^18^	2.8 × 10^19^	2.2 × 10^18^	2.2 × 10^18^	2.8 × 10^18^
VB effective density of states, *N*_*V*_ (1/cm^3^)	1.8 × 10^19^	1 × 10^19^	1.8 × 10^19^	1.8 × 10^19^	1 × 10^19^
Electron mobility, ${\mu }_{n}$ (cm^2^/ V-s)	4.5 × 10^–2^	100	2.10 × 10^–3^	30	12
Hole mobility, ${\mu }_{e}$ (cm^2^/ V-s)	4.5 × 10^–2^	43.9	2.16 × 10^–3^	10	2.8
Shallow uniform acceptor density, *N*_*A*_ (1/cm^3^)	1 × 10^19^	1 × 10^18^	1 × 10^18^	1 × 10^18^	1 × 10^18^
Shallow uniform donor density, *N*_*D*_ (1/cm^3^)	–	–	–	–	–
Total defect density, *N*_*t*_ (1/cm^3^)	1 × 10^15^	1 × 10^15^	1 × 10^15^	1 × 10^15^	1 × 10^15^
Thermal velocity of electrons and holes (cm/s)	1 × 10^7^	1 × 10^7^	1 × 10^7^	1 × 10^7^	1 × 10^7^
Capture cross section of electrons and holes (cm^2^)	2 × 10^–14^	2 × 10^–14^	2 × 10^–14^	2 × 10^–14^	2 × 10^–14^

**Table 3:** Optimization parameters for different hybrid electron transport layer^14–16,27^.

Hybrid ETL	PCBM–SnS_2_	TiO_2_–SnO_2_	PCBM–PCPB
Thickness (nm)	45	50	50
Band gap, *E*_*g*_ (eV)	1.57	3.3	2
Electron affinity, χ (eV)	4	4	3.9
Dielectric permittivity (relative), ε_r_	4.2	9	3.9
CB effective density of states, *N*_*C*_ (1/cm^3^)	2.5 × 10^19^	2.1 × 10^18^	2.5 × 10^21^
VB effective density of states, *N*_*V*_ (1/cm^3^)	2.5 × 10^19^	1.8 × 10^19^	2.5 × 10^21^
Electron mobility, ${\mu }_{n}$ (cm^2^/ V-s)	2.89 × 10^–1^	30	5.5 × 10^–4^
Hole mobility, ${\mu }_{e}$ (cm^2^/ V-s)	2.89 × 10^–1^	15	5.5 × 10^–4^
Shallow uniform acceptor density, *N*_*A*_ (1/cm^3^)	–	–	–
Shallow uniform donor density, *N*_*D*_ (1/cm^3^)	2.4 × 10^17^	2 × 10^20^	3 × 10^17^
Total defect density, *N*_*t*_ (1/cm^3^)	1 × 10^15^	1 × 10^15^	1 × 10^15^
Thermal velocity of electrons and holes (cm/s)	1 × 10^7^	1 × 10^7^	1 × 10^7^
Capture cross section of electrons and holes (cm^2^)	2 × 10^–14^	2 × 10^–14^	2 × 10^–14^

**Table 4:** Parameters for interface layers^12,27^.

Parameters	Perovskite	Hybrid ETL/perovskite interface	Perovskite/HTL interface
Defect type	Neutral	1 × 10^15^	1 × 10^15^
Energetic distribution	Gaussian	Single	Single
Energy levels with respect to Ev (eV)	0.65	0.65	0.65
Characteristic energy (eV)	0.1	0.1	0.1

## Results and discussion

### Study of HTLs, lead perovskite absorbers, and hybrid ETLs

**Figure 2:**
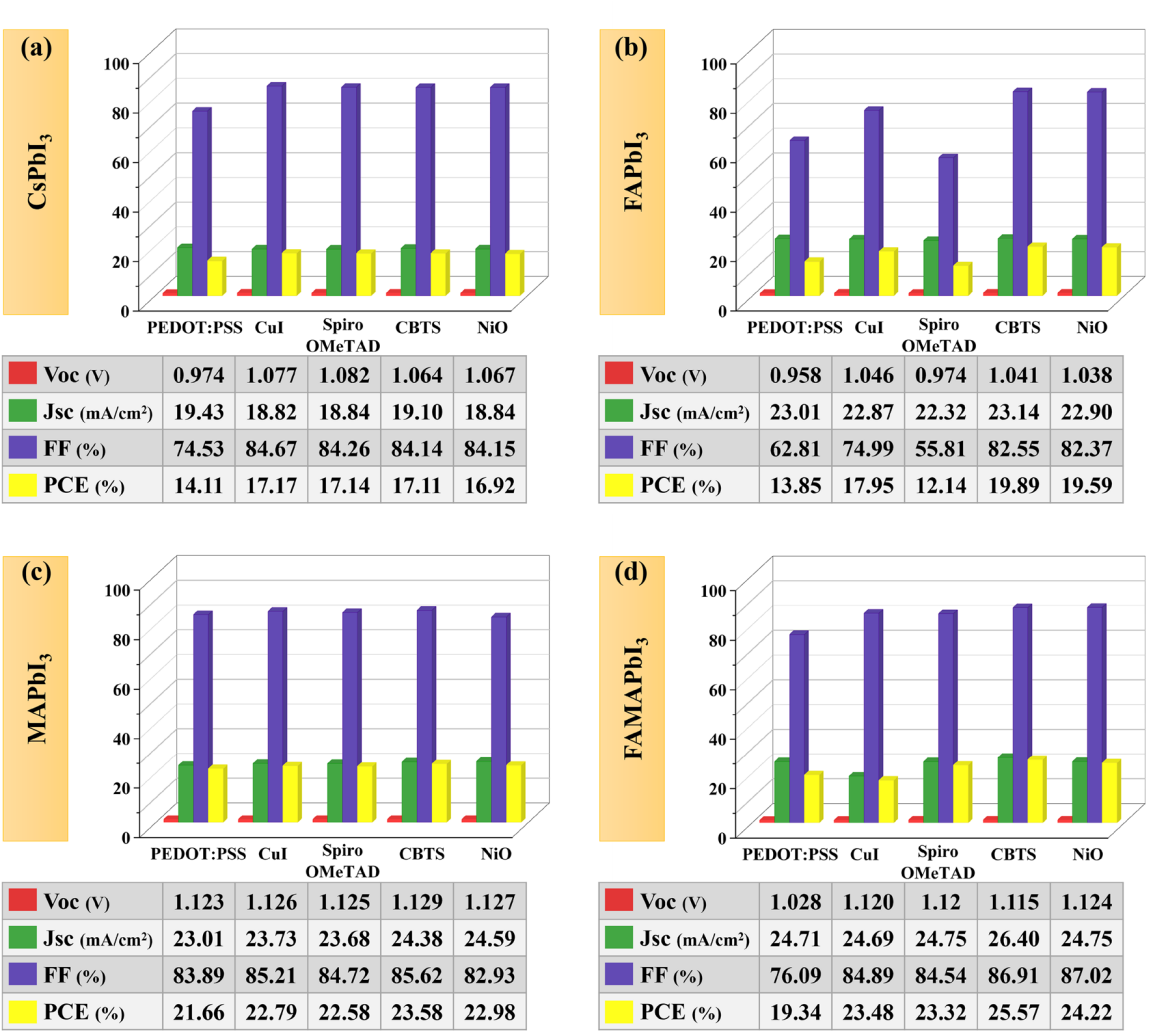
Variation of solar cell parameters, i.e., *Voc, Jsc, FF,* and *PCE* of absorber layers (**a**) CsPbI_3_, (**b**) FAPbI_3_, (**c**) MAPbI_3_, and (**d**) FAMAPbI_3_-based perovskite solar cell devices for different HTLs with a hybrid PCBM–SnS_2_ ETL.

**Figure 3:**
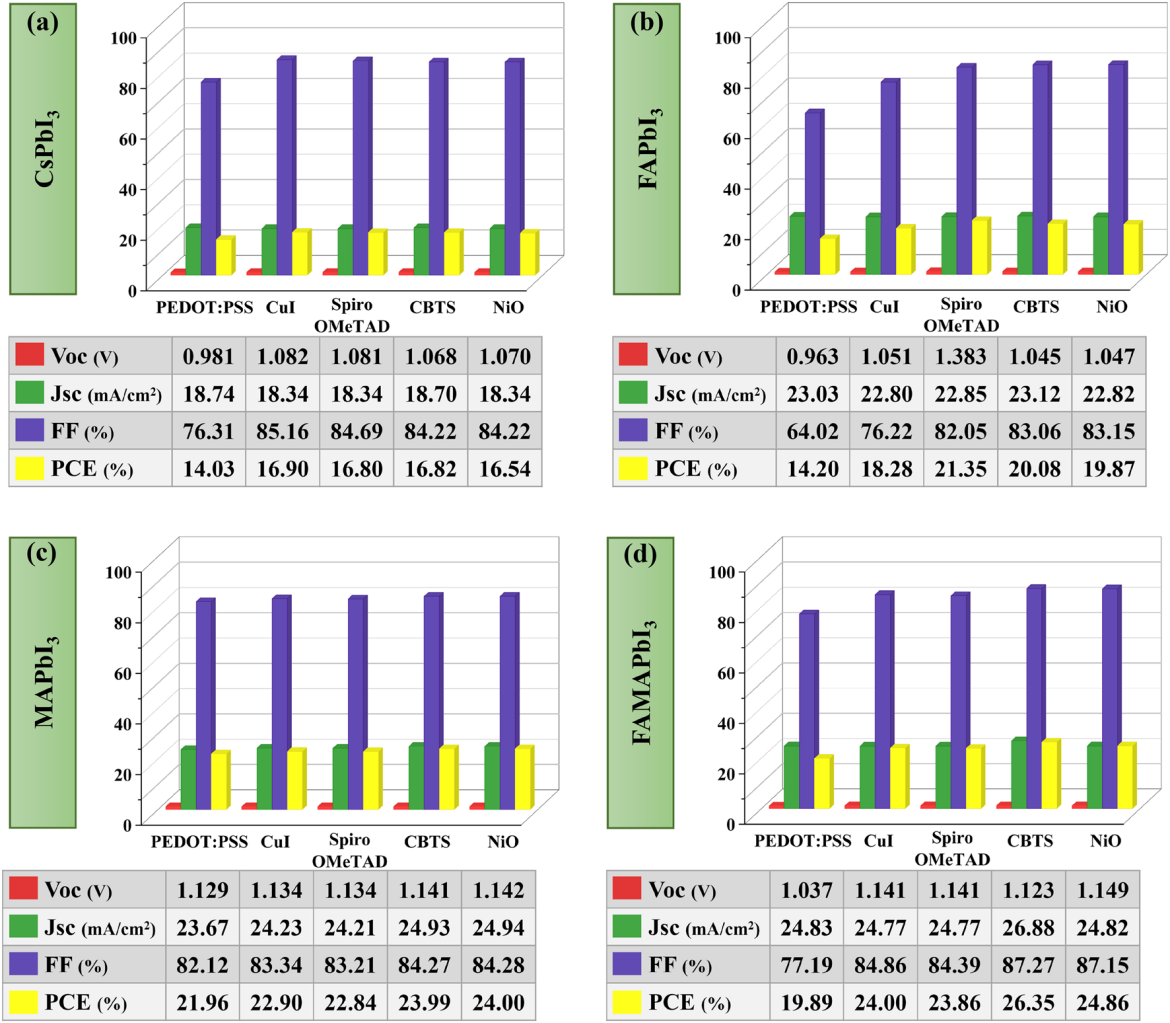
Variation of solar cell parameters, i.e., *Voc, Jsc, FF,* and *PCE* of absorber layers (**a**) CsPbI_3_, (**b**) FAPbI_3_, (**c**) MAPbI_3_, and (**d**) FAMAPbI_3_-based perovskite solar cell devices for different HTLs with a hybrid TiO_2_–SnO_2_ ETL.

**Figure 4:**
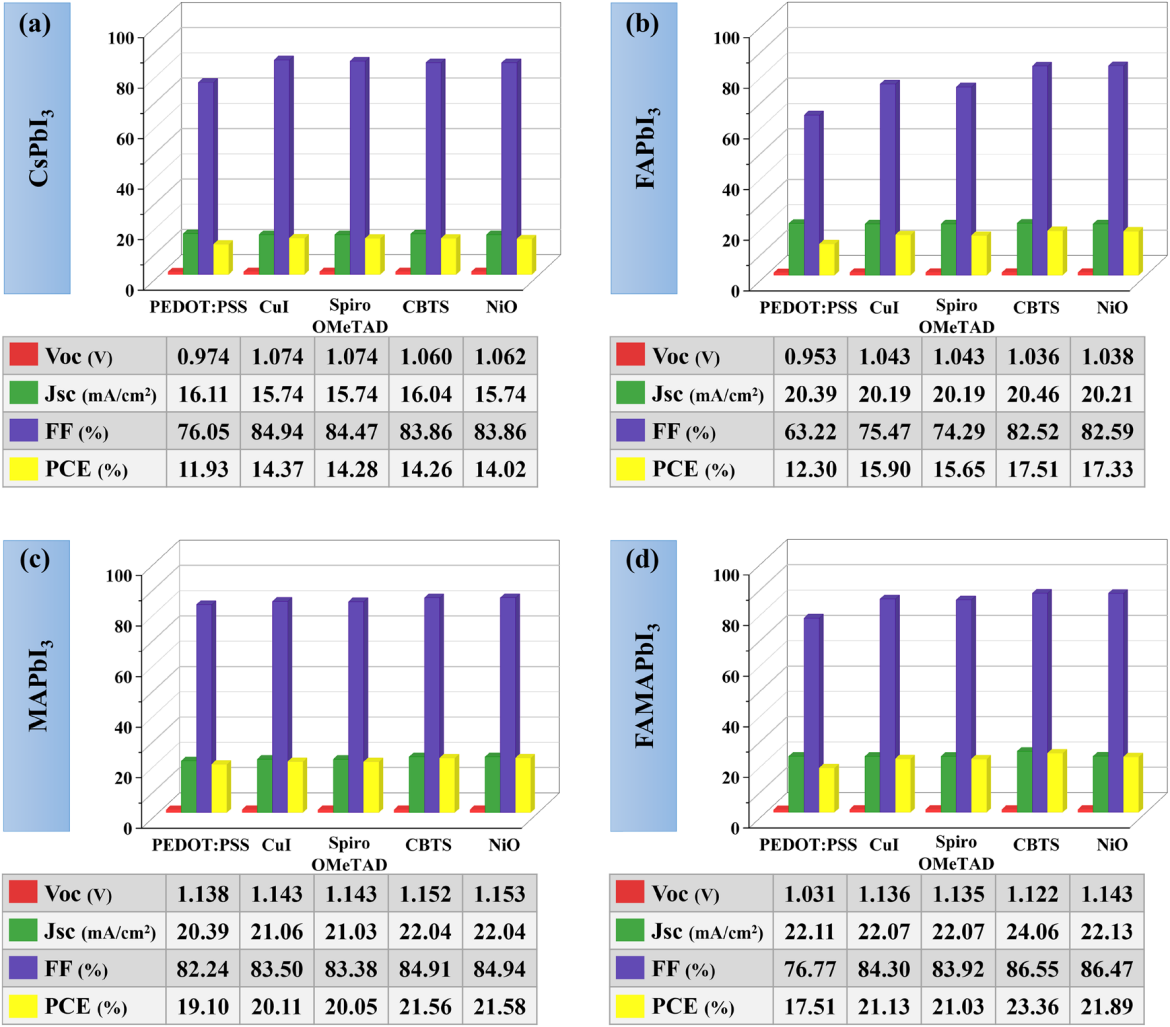
Variation of solar cell parameters, i.e., *Voc, Jsc, FF,* and *PCE* of absorber layers (**a**) CsPbI_3_, (**b**) FAPbI_3_, (**c**) MAPbI_3_, and (**d**) FAMAPbI_3_-based perovskite solar cell devices for different HTLs with a hybrid PCBM–PCPB ETL.

In the device configuration for perovskite solar cells, HTL transmits holes from the perovskite to the back metal contact. Five different HTLs and four different perovskite types have been employed in the simulation program to optimize performance. Figures 2, 3 and 4 illustrates the HTL optimization procedure for four distinct perovskites using various hybrid ETLs. Figure 2a shows that CsPbI_3_ as the absorber layer is optimized with CuI as the HTL compared to the other HTL, while the PCE of this perovskite device configuration is 17.17%, with fixed hybrid PCBM–SnS_2_ ETL. Additionally, FAPbI_3_ as the absorber demonstrated maximum optimization in Fig. 2b using CBTS as the HTL, with a PCE of 19.89%. With a PCE of 23.58%, MAPbI_3_ as the absorber and CBTS as the HTL showed greater optimization than other HTL, as shown in Fig. 2c. Comparing various perovskite absorbers, the absorber FAMAPbI_3_ with CBTS as HTL displayed the highest PCE of 25.57%.

Similar to the fixed hybrid TiO_2_–SnO_2_, Fig. 3 shows that the optimal HTLs for CsPbI_3_, FAPbI_3_, MAPbI_3_, and FAMAPbI_3_ were CuI, Spiro-OMeTAD, NiO, and CBTS. These device combinations' PCEs are 16.90%, 21.35%, 24%, and 26.35%, respectively. According to the results, FAMAPbI_3_ with CBTS as HTL exhibits a high power conversion efficiency of 26.35% with a hybrid ETL made of TiO_2_–SnO_2_.

Using hybrid PCBM–PCPB ETL, Fig. 4 shows that CsPbI_3_, FAPbI_3_, MAPbI_3_, and FAMAPbI_3_ as absorbers achieved maximum optimization with CuI, CBTS, NiO, and CBTS as HTLs compared to other HTLs. These device combinations' PCEs are 14.37%, 17.51%, 21.58%, and 23.36%, respectively. Yet again, the results make it abundantly evident that FAMAPbI_3_ with CBTS as HTL exhibits high power conversion efficiency of 23.36% with PCBM–PCPB as a hybrid ETL.

Comparing various HTL and perovskite absorbers presented in this study, CBTS as HTL and FAMAPbI_3_ as absorbers exhibited the highest performance with different hybrid HTL. Therefore, FAMAPbI_3_ with CBTS is appropriate for optimization across a variety of simulated device architectures. In the following section, optimization is done for the FAMAPbI_3_-based perovskite solar cell with CBTS as HTL.

### Effect of absorber layer thickness on solar cell characteristics

**Figure 5:**
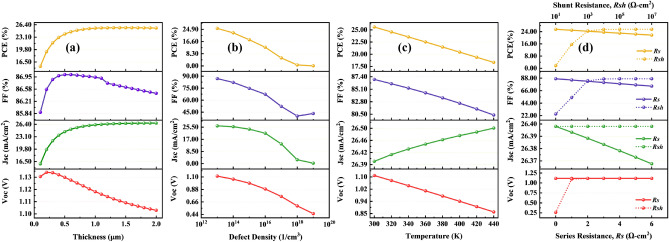
Influence of (**a**) FAMAPbI_3_ absorber thickness, (**b**) total defect density, (**c**) temperature, and (**d**) Parasitic resistance—sheet and shunt resistance on the parameters (Voc, Jsc, FF, and PCE) of a PCBM–SnS_2_ hybrid ETL-based perovskite solar cell.

**Figure 6:**
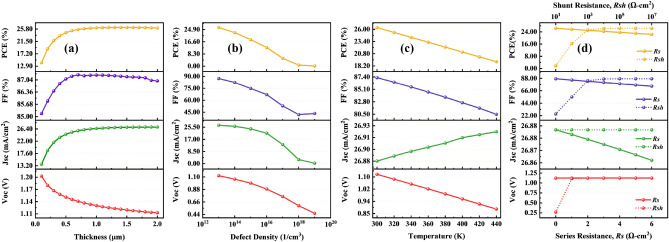
Influence of (**a**) FAMAPbI_3_ absorber thickness, (**b**) total defect density, (**c**) temperature, and (**d**) Parasitic resistance—sheet and shunt resistance on the parameters (Voc, Jsc, FF, and PCE) of a TiO_2_–SnO_2_ hybrid ETL-based perovskite solar cell.

**Figure 7:**
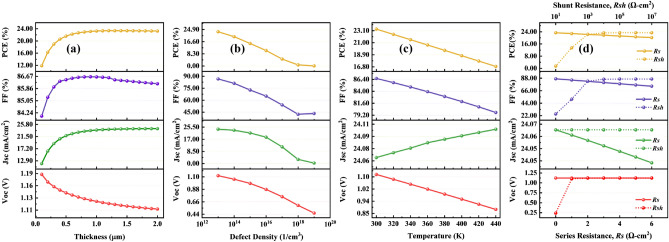
Influence of (**a**) FAMAPbI_3_ absorber thickness, (**b**) total defect density, (**c**) temperature, and (**d**) Parasitic resistance—sheet and shunt resistance on the parameters (Voc, Jsc, FF, and PCE) of a PCBM–PCPB hybrid ETL-based perovskite solar cell.

In a perovskite solar cell, an absorber layer plays a crucial role in absorbing light and converting it into electrical energy. The absorber layer, made of perovskite material, has high absorption coefficients, allowing it to efficiently absorb incident photons over a broad range of wavelengths and generate charge carriers ^29^. To create the most electron–hole pairs and absorb the most photons while minimizing reflection and absorption losses, the absorber layer should be placed at the ideal thickness. The optimum thickness of an absorber layer for a solar cell varies depending on the material used for the layer. It typically ranges from several hundred nanometers to a few micrometers. When the absorber layer thickness increases, the stability increases, and the longer wavelength of light will produce a good amount of electron–hole pair generation^12^. However, the thicker perovskite layers may decrease the fill factor due to recombination and resistive losses^30^. Reducing the thickness of the absorber layer also leads to reduced fill factor and efficiency. This is because the depletion layer becomes very close to back contact. The back contact will capture more electrons for recombination and leave behind fewer electrons to participate in the generation process^31^. As a result, a solar cell's performance and outcome can be greatly impacted by changes in the perovskite layer. To maximize photon absorption, it should be properly chosen, and it should not be too big to reduce reverse saturation current. When the absorber layer thickness varied in this study from 100 to 2000 nm, we looked at how it affected the output parameters while keeping the other device characteristics constant. Figures 5a, 6a, and 7a show variations in solar cell parameters by changing the values of the thickness of the absorber layer. It can be noticed in Figs. 5a, 6a, and 7a, that the PCE and the Jsc values increase with increasing FAMAPbI_3_ thickness for all structures with different hybrid ETLs. The increase in PCE is due to the absorption of more photons by the absorber layer, which consequently generates more charge carrier concentration. However, the PCE of hybrid ETL (PCBM–SnS_2_, TiO_2_–SnO_2,_ and PCBM–PCPB) based solar devices have slightly decreased after 1500 nm, 1300 nm, and 1200 nm due to an increase in recombination of charge carriers within the material. The Voc and the FF decreased with increasing the perovskite layer thickness due to increased series resistance and internal power dissipation of the perovskite structure. The decrease in Voc maybe also due to the increase in carrier recombination rate and dark saturation current I_0_. The following equation can express this effect ^32^,

4 $${\text{Voc = }}\frac{{{\text{nkT}}}}{{\text{q}}}{\text{ln}}\left( {\frac{{{\text{I}}_{{\text{L}}} }}{{{\text{I}}_{{0}} }}{ + 1}} \right)$$

where I_L_ represents photogenerated current, n denotes the ideality factor and kT/q is the thermal voltage. A similar trend is shown. Therefore, the optimal thickness of FAMAPbI_3_ associated with hybrid ETLs, PCBM–SnS_2_, TiO_2_–SnO_2,_ and PCBM–PCPB are fixed at 1500 nm, 1300 nm, and 1200 nm for further calculation.

### Effect of absorber total defect density (***N***_***t***_) on solar cell device

The absorber layer of a solar cell is where activities like carrier generation, carrier recombination, and charge transport take place, hence the quality of the absorber material is crucial. So, the quality of the absorber material, which is dependent on the defect density, has a substantial impact on the characteristics of solar cells^18^. The defect density in perovskites refers to the number of defects or impurities present in a perovskite material. Various defects include vacancies, interstitials, Frenkel and Schottky^17,33^. In the context of perovskites, defects can significantly affect the material's optical, electrical, and thermal properties, impacting solar cell performance and efficiency^18^. The Gaussian distribution method can be used to understand the defect density of perovskites in solar cells by characterizing the distribution of defect states in the energy bandgap of the material. In this method, the energy bandgap of the perovskite material is divided into discrete energy levels and the probability of finding an electron or hole in each energy level is calculated using Gaussian distributions^34^. The following are the equations associated with Gaussian distribution models,

5 $${g}_{D}\left(E\right)= {G}_{Md}\mathrm{exp}[\frac{{-\left(E-{E}_{pkd}\right)}^{2}}{2{\sigma }_{d}^{2}}]$$

6 $${g}_{A}\left(E\right)= {G}_{Ma}\mathrm{exp}[\frac{{-\left(E-{E}_{pka}\right)}^{2}}{2{\sigma }_{a}^{2}}]$$

where *σ*_*d*_ and *σ*_*a*_ are the standard energy deviations of the Gaussian acceptor and donor levels, ${G}_{Md}$ and ${G}_{Ma}$ are effective defect densities, ${E}_{pkd}$ and ${E}_{pka}$ are the donor and acceptor peak energy positions measured from *Ec* and *Ev*. Overall, the Gaussian distribution method is a useful tool for understanding the impact of defects on the performance of perovskite solar cells, and for developing strategies to improve the efficiency and stability of these devices.

We simulate the device's performance by changing the perovskite defect density from 10^13^ to 10^19^ cm^−3^ to investigate the impact of the absorber's defect density on the device's performance. Figures 5b, 6b, and 7b represent the variation of device parameters Voc, Jsc, FF, and PCE with a simultaneous variation of defect density for different ETLs used in our analysis. The charts show that all the hybrid ETLs virtually have a similar character to the way that the device parameters vary. The parameters' range of values is where there is the sole difference. As the total defect density increases, the cell performance deteriorates. The power conversion efficiency of the PSCs was drastically reduced from 25.57 to 0.05%, 26.35 to 0.06%, and 23.36 to 0.06% for PCBM–SnS_2_, TiO_2_–SnO_2,_ and PCBM–PCPB, respectively as the defect density is increased. Other parameters such as FF, Jsc, and Voc also decrease with an increase in defect density. A rapid reduction in photovoltaic characteristics with increased defects is due to increased recombination due to the generation of pinholes^7^. Additionally, it causes the diffusion length to decrease, which lowers the number of carriers that may reach the contacts. High defect density can also affect the stability of perovskite solar cells, leading to rapid degradation and short device lifetimes^12^. With increasing defects, the number of traps also increases, causing carriers to be trapped and reducing their mobility and efficiency^35^. The maximum power efficiency obtained in the numerical simulation for all hybrid ETLs was at *N*_*t*_ = 10^13^ cm^−3^. Expecting a very low defect density in any material is impracticable since it is exceedingly challenging to synthesize a material with a low density. We continue to use the optimum absorber defect density of 10^13^ cm^−3^ for further simulations.

### Effect of temperature on the device

Solar cells are widely used in outdoor applications where the device is illuminated by continuous sunlight. If a solar panel is not properly ventilated or insulated, heat from the surrounding environment can increase the temperature^12^. A solar cell's temperature may also rise as a result of the internal conversion of solar energy into electrical energy. This process produces heat, which can raise the temperature, increase stress and strain on structures, and have an adverse effect on the efficiency and power output of the solar cell by causing interfacial flaws, disorder, and poor interconnection between layers^36^. On the other hand, low temperatures can increase the cell’s resistance, reducing its efficiency. Therefore, the temperature is one of the most crucial factors affecting solar cell performance. In this study, we examine the relationship between temperature and cell performance by simulating the device’s temperature ranging between 300 and 440 K, maintaining the overall parameters constant. Figures 5c, 6c, and 7c show the influence of temperature on the key parameters of perovskite solar cells. It is observed from the plot that the nature of variation of the device parameters is almost similar for all three hybrid ETL-based perovskite structures. The power conversion efficiency linearly reduced from 25.57 to 18.33%, 26.35 to 19.08%, and 23.36 to 16.87% for PCBM–SnS_2_, TiO_2_–SnO_2,_ and PCBM–PCPB, with increasing temperature. In addition to PCE, FF has also decreased with increasing temperature for all optimum structures due to the degradation of the perovskite material, rise in recombination rate, and saturation current, leading to decreased stability and a shorter lifespan^37,38^. More interfacial defects, together with an increase in series resistance and a decrease in the exciton diffusion length, are the cause of the fall in Voc as temperature increases^22^. In contrast, the Jsc was affected slightly by the increase in temperature due to the bandgap reduction and creation of a greater number of electron–hole pairs^39^. However, the range of change for all optimal device designs with temperature variation is quite small and appears to be practically constant. The mobility of charge carriers in the perovskite material is also affected by temperature, leading to changes in the current–voltage characteristics of the PSC^36^. In the variation of temperature, the maximum performance of the perovskite structures is perceived at 300 K. Therefore, 300 K is considered the optimum temperature for further simulations of hybrid ETL-based perovskite solar cells.

### Effect of change in series and shunt resistances on the device

The series (R_s_) and shunt resistances (R_sh_) generated due to the connections between layers of the solar cell, contacts with metal, and flaws in manufacturing have a significant impact on the device performance, especially on the fill factor (FF) and short circuit current (Jsc)^40^. These parasitic resistances mainly control the slopes and shape of the current–voltage characteristics^41^. According to the Shockley diode model, the following equations give the current/voltage relationship with sheet and shunt resistances during illumination^42^.

7 $${J}_{sc}={J}_{I}- {J}_{0}\left[exp\left(\frac{{q}_{e}\left(V-J{R}_{S}\right)}{nkT}\right)-1\right]-\frac{V-J{R}_{s}}{{R}_{sh}}$$

8 $${V}_{oc}=\left(\frac{nkT}{{q}_{e}}\right)ln\left\{\frac{{J}_{I}}{{J}_{0}}\left(1-\frac{{V}_{oc}}{{J}_{I}{R}_{sh}}\right)\right\}$$

where *J*_*sc*_ is the current produced by the short circuit, ${q}_{e}$ is the elementary charge, *J*_*I*_ is the electric photocurrent, *J*_0_ is the density of reverse saturation current, *V* is the output voltage, *k* is the Boltzmann constant, *n* is the ideality factor of the diode, and *T* is the ambient temperature.

The sources of sheet resistance in PSCs include internal resistances, interface barriers, charge-collecting interlayers, and metal-based electrodes. Shunt resistance, on the other hand, comes from leakage channels such as recombination losses and pinholes in the photoactive layer. Resistance falls off as the series progresses^38^. As a result, with greater values of series resistance, the short circuit current also starts to decrease. The Voc is almost not influenced by the R_s_ because the total current flow through the solar cell and consequently the series resistance is zero. Therefore, a solar cell's PCE is low when its series resistance is high^20^. To account for this change in device characteristics for the perovskite active materials, the variation in series resistance was investigated. The effect of R_s_ varied from 0 to 6 Ω cm^2^ in the three (ITO/Hybrid ETL/FAMAPbI_3_/CBTS) devices as shown in Figs. 5d, 6d, and 7d. In the variation of R_s_, PCE was significantly decreased for all three hybrid ETL-based perovskite device structures. The PCE value of PCBM–SnS_2_ as a hybrid ETL-based perovskite device structure decreased from around 25.6 to 21.76%. In contrast, with increasing sheet resistance, TiO_2_–SnO_2_ as a hybrid ETL-based device structure decreased from 26.35 to 22.36%. And PCBM–PCPB hybrid ETL-associated solar cell structure showed almost 23.36 to 20.18% PCE, respectively, with the increased sheet resistance. While the FF value of all hybrid ETL-associated solar cells showed a greater value, it likewise declined as R_s_ increased. All hybrid ETL systems had a very slight drop in Jsc value as R_s_ increased. The Voc performance showed the constant value for all hybrid ETL-based device configurations with increasing R_s_. Thus, it is verified that R_s_ variation does not impact the Voc parameter for the studied device configurations. The best performance of the device configurations is observed when no sheet resistance is present (i.e., 0 Ω cm^2^). Therefore, it is considered the optimized value of sheet resistance for further simulation.

Further, the effect of device parameters with R_sh_ variation is visually represented in Figs. 5d, 6d, and 7d, where R_sh_ varies from 10^1^ to 10^7^ Ω cm^2^ for all three optimum solar cell structures. The values of device parameters showed a similar pattern with increasing shunt resistance (R_sh_) for all three structures. The PCE and FF parameters increased rapidly from 10^1^ to 10^3^ Ω cm^2^ and then maintained constant value with increasing R_sh_. Similarly, Voc increased from 10^1^ to 10^2^ Ω cm^2^ and maintained a constant value for all device configurations. While Jsc values remained constant (no change) for all values of shunt resistances. Therefore, the enhancement in devices’ efficiency can be explained by the FF increase. The optimized shunt resistance value is observed at 10^1^ Ω cm^2^ where the device performance is maximum for all three hybrid ETL-based perovskite solar cells.

### Effect of capacitance and Mott–Schottky on the device

**Figure 8:**
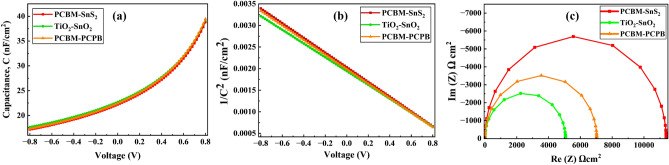
A comparison of (**a**) Capacitance (C), (**b**) Mott–Schottky (1/C^2^), and (**c**) Nyquist plot for three hybrid ETL-based perovskite solar devices.

The effect of capacitance on solar cells is related to the storage and release of electrical charge within the cell, affecting its overall efficiency and performance. When a solar cell is exposed to light, the amount of charge stored in its capacitance can change, leading to changes in the cell's voltage and current output^43^. The Mott–Schottky effect, on the other hand, refers to the relationship between the electronic properties of the perovskite/electrode interface, including the built-in potential and the density of states. This effect can also impact the performance of a solar cell by affecting the flow of current through the cell. This can have a significant impact on the performance of perovskite solar cells, as the built-in potential affects the open-circuit voltage (Voc) of the cell, which is one of the key parameters determining its overall efficiency^27,43^. The Mott–Schottky effect can also affect the electron transport properties in the perovskite layer, which can impact the photocurrent generation and the overall power conversion efficiency of the cell. Overall, both capacitance and the Mott-Schottky effect play important roles in the performance and efficiency of solar cells, and understanding these effects is crucial for optimizing the design and operation of solar power systems. Figure 8a, b show the plots of capacitance per unit area (C) and Mott–Schottky (M–S) plots with a bias voltage (V), respectively, for three optimized hybrid ETL-based perovskite solar cells. It is observed that all hybrid ETL-based devices represent the independent voltage capacitance because of the saturation of depletion layer capacitance^27^. The slope of the Mott–Schottky plot gives the carrier density of the charge-selective layer, while the intercept gives the built-in potential. The carrier density of PCBM–SnS_2_, TiO_2_–SnO_2,_ and PCBM–PCPB hybrid ETL based perovskite solar cell is found to be 1.7 × 10^15^, 1.6 × 10^15^ and 1.68 × 10^15^ cm^−3^. While the x-intercept that gives the built in potential is as follows: 1.19 V, 1.21 V and 1.18 V. Among the three hybrid ETL based device structure, TiO_2_–SnO_2_ has shown better performance.

### Electrochemical impedance spectroscopy analysis of hybrid ETLs

To comprehend the function of the hybrid ETL, electrochemical impedance spectroscopy (EIS) investigations have been conducted in this section. This technique widely characterizes perovskite solar cells. It is used to check the effect of impedance responses of material (ETL in this case) against a wide frequency range (1 Hz to 1 MHz). The data is plotted on a Nyquist plot, which can provide information about the system's capacitance, resistance, and phase angle^44^. The two resistive characteristics of solar cells are the R_s_ (series resistance), which is related to the resistance of the materials and back/front contacts, and the R_rec_ (recombination resistance), which is inversely related to the recombination rate of the carriers. High recombination resistance allows for increased collection and utilization of the electrons and holes, leading to higher efficiency and performance of the solar cell. The R_rec_ and Rs can be calculated by measuring the point where the curve and x-axis connect at high and low frequencies^44^.

Three perovskite solar cells with PCBM–SnS_2_, TiO_2_–SnO_2,_ and PCBM–PCPB hybrid were analyzed using EIS. The impedance spectra in Fig. 8c show three semicircles in the Nyquist plot under short circuit conditions. The semi-circle is formed because the imaginary component of impedance, which is related to capacitance, is proportional to the sine of the phase angle, while the real component, which is related to resistance, is proportional to the cosine of the phase angle. As the frequency of a system increases, the phase angle changes, and this change is reflected in the shape of the semi-circle. The center of the semi-circle represents the "critical point" of the system, and the radius of the semi-circle is proportional to the magnitude of the capacitance. A large semi-circle radius indicates a large capacitance, while a small radius indicates a small capacitance. That is larger the diameter of the semicircle, the higher the recombination resistance confirms a better p–n junction resulting in ameliorated device performance^44^. It is observed that a large semicircle is formed in the impedance spectrum for PCBM–SnS_2_ substituted cell when compared with the other two hybrid ETLs. Therefore, we can conclude that the solar cell containing PCBM–SnS_2_/FAMAPbI_3_ junction exhibits higher recombination resistance reducing electron capturing by defect traps, suppressing interface recombination, and thereby improving efficiency.

### Study of conduction band offset (CBO)

Due to the formation of optimal energy levels of the conduction band between the ETL and the absorber layer in perovskite solar cells, band alignment significantly contributes to efficiency improvement. The Conduction Band Offset (CBO) between the absorber and ETL considerably affect the transport of carriers, and they have been accepted in three shapes: cliff shape, flat shape, and spike-like shape. The following equation defines the conduction band offset^21^,

9 $${\text{CBO }} = \, \chi {\text{ (PEROVSKITE) }} - \, \chi {\text{ (ETL)}}$$

where χ refers to electron affinity.

**Figure 9:**
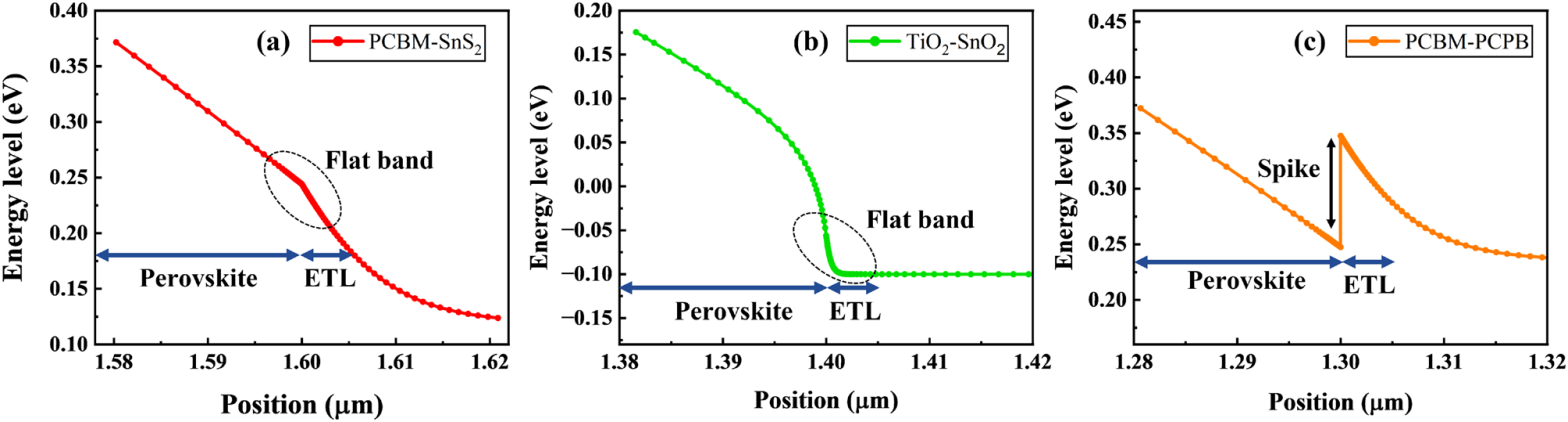
Energy levels of the conduction band (Ec) for (**a**) FAMAPbI_3_/PCBM–SnS_2_, (**b**) FAMAPbI_3_/TiO_2_–SnO_2_, and (**c**) FAMAPbI_3_/PCBM–PCPB structures.

According to the above equation, conduction band offset values for each hybrid ETL-associated perovskite have been calculated and listed in Table 5. When the energy difference is zero, as in the case of the CBO of FAMAPbI_3_/PCBM–SnS_2_ and FAMAPbI_3_/TiO_2_–SnO_2_ (see Fig. 9a, b), then the shape is nearly flat, which means that there is no band offset and consequently no barrier for the transport of charge carriers (generated electrons or holes).

**Table 5:** Conduction band offset values for each hybrid ETL-based perovskite.

Perovskite/ETL	Electron affinity of Perovskites (eV)	Electron affinity of hybrid ETLs (eV)	CBO (eV)	Band alignment
FAMAPbI_3_/PCBM–SnS_2_	4	4	0	Flat
FAMAPbI_3_/TiO_2_–SnO_2_	4	4	0	Flat
FAMAPbI_3_/PCBM–PCPB	4	3.9	+ 0.1	Spike

The band alignment of the absorber/ETL exhibits a spike-like appearance for tiny positive CBO values as in the case of FAMAPbI_3_/PCBM–PCPB (see Fig. 9c), preventing recombination at the interface. When CBO is negative, the absorber/ETL band alignment is cliff-like, facilitating increased and faster recombination at the interface^21^. A significant reduction in Jsc and FF is associated with large positive CBO values. This reduction is owing to the formation of a strong barrier against electrons that are generated by light.

### J–V and QE characteristics

**Figure 10:**
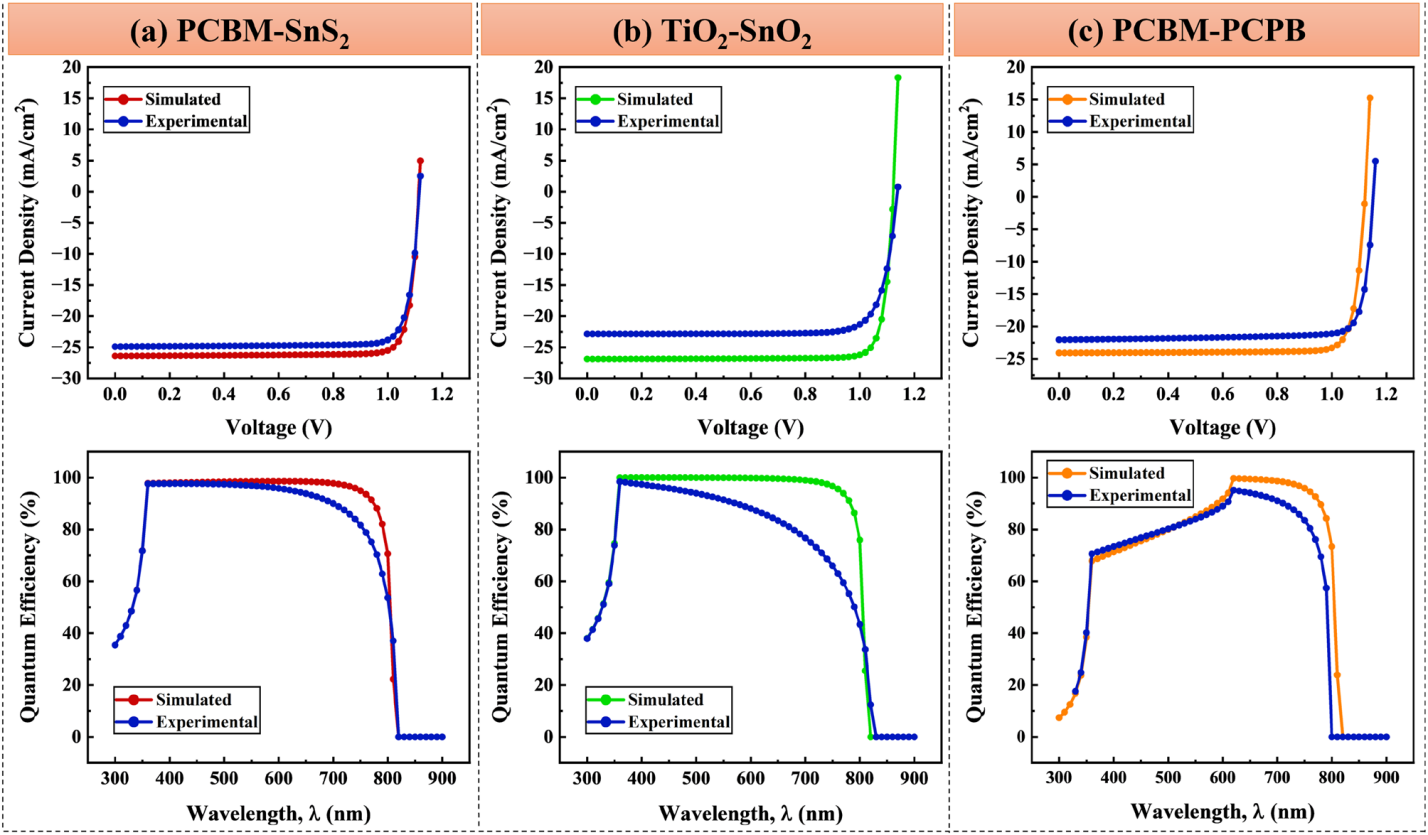
J–V and QE-wavelength curves were obtained for both simulation and experimental work.

Quantum efficiency curves and current density–voltage characteristics (J–V) are essential for analyzing solar cell performance^12^. According to the study, the added ETLs that increase device stability and durability are responsible for the increased efficiencies of FAMAPbI_3_ solar cells. For each device structure having PCBM–SnS_2_, TiO_2_–SnO_2,_ and PCBM–PCPB as hybrid electron transport materials, the comparison between the simulated and experimental results is presented in Fig. 10 and the value of device parameters is summarized in Table 6. According to the results, in the FAMAPbI_3_ solar cell proposed with different ETLs, the Jsc, FF, and PCE are increased compared to the reference solar cell. Thus, hybrid ETLs are highly efficient to improve the performance of perovskite solar cells with high stability.

**Table 6:** Solar cell parameters exhibit superior agreement with experimental work.

Hybrid ETL	Voc (V)	Jsc (mA/cm^2^)	FF (%)	PCE (%)	References
PCBM–SnS_2_	1.11	26.4	86.91	25.57	This work
TiO_2–_SnO_2_	1.12	26.88	87.27	26.35	This work
PCBM–PCPB	1.12	24.06	86.55	23.36	This work
PCBM–SnS_2_	1.06	22.70	82.87	19.95	^14^
PVP–SnO_2_	1.11	22.43	76.20	18.98	^13^
IGZO/SnO_2_–PCBM	0.92	22.98	61	12.56	^45^
TiO_2_–SnO_2_	1.18	22.53	79.18	21.02	^15^
TiO_2_–WO_x_	1.16	22.89	79.3	20.83	^46^
PCBM–PCPB	1.08	22.51	76.01	18.48	^16^

**Figure 11:**
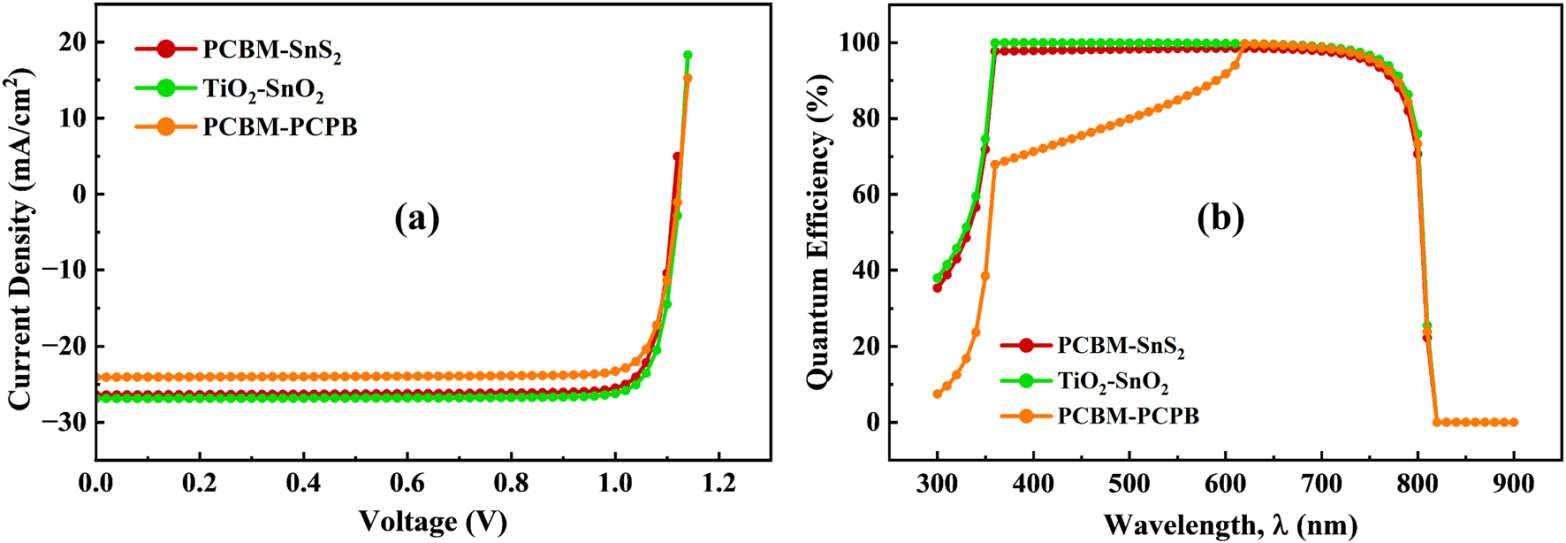
(**a**) J–V characteristics and (**b**) QE curve of the optimized FAMAPbI_3_ perovskite solar cells with CBTS as HTL for different hybrid ETLs (PCBM–SnS_2_, TiO_2_–SnO_2,_ and PCBM–PCPB).

Figure 11a shows the J–V characteristics with an identical pattern of the studied three solar cells. While Fig. 11b shows the quantum efficiency curve against the wavelength ranging from 300 to 900 nm covering the visible and near infrared spectrum. According to Fig. 11a, it is noticed that TiO_2_–SnO_2_ as an ETL-associated PSC showed better performance at about 26.88 mA/cm^2^ as current density, while the Voc is 1.12 V. Additionally, due to a large increment in Jsc, TiO_2_–SnO_2_ as an ETL based shows an enhanced transmission of the short wavelength photons. As shown in Fig. 11b, the proposed solar cell has an improved external quantum efficiency compared to the other two device structures, demonstrating excellent photon utilization.

## Conclusion

Various hybrid perovskite solar cells with the perovskite layer (CsPbI_3_, FAPbI_3_, MAPbI_3,_ and FAMAPbI_3_), and their performance are analyzed using the solar simulator software. PCBM–SnS_2_, TiO_2_–SnO_2,_ and PCBM–PCPB were used as hybrid ETLs, PEDOT: PSS, CuI, Spiro-OMeTAD, CBTS, and NiO were used as HTLs. The CBTS and FAMAPbI_3_ as HTL and absorber have depicted the best performance with the hybrid ETLs. The influences of different factors on the performance of perovskite solar cells have been studied to optimize the device configuration and improve the efficiency of perovskite solar cells with hybrid ETLs. The highest efficiency was achieved via cell configuration ITO/TiO_2_–SnO_2_/FAMAPbI_3_/CBTS/Metal with Voc = 1.12 V, Jsc = 26.88 mA/cm^2^, FF = 87.27%, and PCE = 26.35%. Other hybrid ETL-associated perovskites also exhibited nearly identical effects on the studied factors. Hence, the reported perovskite solar cells with a hybrid electron transport layer provide a viable path to realizing environmentally benign, low-cost, highly stable, and efficient PSC.

## Data Availability

The manuscript data that support the findings of this study are available from the corresponding authors upon reasonable request.

## Code availability

The manuscript used code that supports the electrical 1D SCAPS simulations are available from the corresponding authors upon reasonable request.
